# Comparative study of transvaginal ultrasound and magnetic resonance imaging in the diagnosis and classification of cesarean scar pregnancy

**DOI:** 10.1590/1806-9282.20250952

**Published:** 2026-01-09

**Authors:** Qinghu Li, Ao Yin, Meng Zhang, Sha Tian

**Affiliations:** 1 Huazhong University of Science and Technology, Tongji Medical College, Wuhan Children's Hospital, Wuhan Maternal and Child Healthcare Hospital, Department of Ultrasound for Women and Children – Wuhan, China.

**Keywords:** Ultrasound, Magnetic resonance imaging, Scar, Pregnancy, Classification

## Abstract

**OBJECTIVE:**

Cesarean scar pregnancy is a rare form of ectopic pregnancy in which the fertilized egg implants within the scar of a previous cesarean section. With the rising global cesarean delivery rate, the incidence of cesarean scar pregnancy has increased, posing serious risks such as massive hemorrhage and uterine rupture. To evaluate and compare the diagnostic and classification accuracy of transvaginal sonography and magnetic resonance imaging in the detection of cesarean scar pregnancy.

**METHODS:**

This retrospective study included 102 patients clinically suspected of cesarean scar pregnancy between January 2023 and May 2024. All patients underwent both transvaginal sonography and magnetic resonance imaging. Using final clinical and pathological diagnoses as the reference standard, the sensitivity, overall diagnostic accuracy, and classification accuracy of transvaginal sonography and magnetic resonance imaging were compared.

**RESULTS:**

Magnetic resonance imaging demonstrated significantly higher sensitivity, diagnostic accuracy, and classification accuracy than transvaginal sonography (p<0.05). Compared with transvaginal sonography, magnetic resonance imaging also showed superior detection rates for intrauterine hemorrhage, villous invasion of the myometrium, and invasion of the bladder and/or cervix, with statistically significant differences (p<0.05).

**CONCLUSION:**

Magnetic resonance imaging demonstrates superior diagnostic and classification accuracy compared to transvaginal sonography in evaluating cesarean scar pregnancy. As a valuable adjunct to ultrasound, magnetic resonance imaging facilitates more accurate diagnosis and individualized treatment planning, thereby reducing the risks of severe complications such as massive hemorrhage, hysterectomy, and maternal mortality in women of reproductive age.

## INTRODUCTION

Cesarean scar pregnancy (CSP) is a rare form of ectopic pregnancy in which the fertilized ovum implants within the fibrous tissue of a previous cesarean section scar. Globally, the incidence of CSP has been rising, paralleling the increasing rate of cesarean deliveries^1^. In China, where cesarean section rates are particularly high, CSP has become an increasingly recognized clinical concern. Early-stage CSP often lacks specific symptoms and may be easily overlooked. As the condition progresses, it can result in severe complications such as uterine rupture, massive hemorrhage, and even maternal mortality^2^.

Currently, no standardized treatment protocol exists for CSP. Management strategies include systemic or local drug therapy, uterine artery embolization, intrauterine curettage, or surgical evacuation of pregnancy tissue—sometimes in combination^3^. Early and accurate diagnosis with appropriate classification is crucial for selecting optimal treatment modalities, improving outcomes, and reducing complications.

The diagnostic approach to CSP typically involves clinical history, imaging evaluation, and in some cases, pathological examination. Intraoperative exploration remains the diagnostic gold standard but is not widely adopted due to its invasiveness^4^. Thus, noninvasive imaging plays an increasingly vital role in early detection. Transvaginal sonography (TVS) is considered the first-line modality due to its ability to assess the gestational sac's location, the thickness of the myometrium at the scar site, and perigestational blood flow. It can also evaluate the relationship between the scar and adjacent organs such as the bladder^5^. However, not all CSP cases display classic sonographic features. In some instances, the gestational sac may be indistinct or display minimal vascularity, increasing the risk of misdiagnosis. Diagnosis is most accurate between the 6th and 7th weeks of gestation; beyond this window, identification becomes more challenging.

Magnetic resonance imaging (MRI), known for its excellent soft tissue contrast and multiplanar capability, has demonstrated efficacy in diagnosing various gynecological conditions^6^. MRI can delineate the spatial relationship between the gestational sac and uterine scar and accurately measure myometrial thickness, providing important supplementary diagnostic information.

This study aims to compare the diagnostic and classification accuracy of TVS and MRI in patients with CSP, evaluate their complementary roles, and guide clinicians in formulating individualized and effective treatment strategies.

## METHODS

### General information

This retrospective study included 102 patients clinically suspected of having CSP between January 2023 and May 2024. The patient age ranged from 20 to 45 years (mean: 33.96±4.08). The number of pregnancies ranged from 2 to 9 (mean: 3.96±1.44), and cesarean sections ranged from 1 to 3 (mean: 1.38±0.55). The interval since the last cesarean section ranged from 1 to 20 years (mean: 6.54±3.89), and amenorrhea duration ranged from 32 to 91 days (mean: 50.23±12.10). All patients had a history of amenorrhea; 61 reported irregular vaginal bleeding, 13 had lower abdominal pain, and 38 were asymptomatic.

Inclusion criteria: prior cesarean section, early pregnancy with intent to terminate, complete clinical data, and both TVS and MRI performed.

Exclusion criteria: contraindications to TVS or MRI, contrast agent allergy, or inability to cooperate with imaging procedures.

### Inspection method

(1) TVS Examination: Transvaginal ultrasound was performed using Voluson E10 or E8 systems (GE, USA). The probe was used to assess uterine size, shape, and gestational sac implantation site. Particular attention was given to the gestational sac's location relative to the cesarean scar, cervical canal, and uterine cavity. The thickness of the myometrium at the scar site and perigestational blood flow were evaluated using color Doppler flow imaging (CDFI). An experienced sonographer (>5 years) interpreted the images based on the 2016 Expert Consensus on CSP diagnosis, which includes: (1) absence of a gestational sac in the uterine cavity and cervical canal; (2) gestational sac located at the cesarean scar site; (3) gestational sac high-velocity, low-resistance blood flow on CDFI; and (4) thinning or discontinuity of the anterior lower uterine myometrium. CSP was classified into three types based on gestational sac location and myometrial thickness: Type I—partial implantation at scar, myometrial thickness >3 mm; Type II—partial implantation, thickness ≤3 mm; Type III—complete implantation with outward bulging toward bladder, thickness ≤3 mm or disrupted continuity.

(2) MRI Examination: MRI was conducted using a PHILIPS 3.0T scanner. Axial, sagittal, and coronal T1-weighted image (T1WI), T2-weighted image (T2WI), and T2WI-SPAIR (Spectral Attenuated Inversion Recovery) sequences were acquired (4 mm slice thickness, 1.2 mm gap), covering L5 to the symphysis pubis. Two radiologists (>10 years’ experience) independently reviewed images, resolving disagreements through consultation. Evaluated parameters included gestational sac morphology, signal characteristics, implantation depth, myometrial thickness, and relationships with the scar and adjacent organs.

### Observation indicators

Based on pathological findings, a comparative analysis was performed to evaluate the diagnostic efficacy of MRI and TVS in diagnosing CSP. Additionally, the detection rates of different CSP types by both imaging modalities were compared.

### Statistical analysis

Data were analyzed using the Statistical Package for Social Sciences (SPSS) 20.0 statistical software (IBM, Armonk, NY, USA). For quantitative data that followed a normal distribution, the mean±standard deviation ( $\bar{\text{χ}}\pm \text{s}$ ) was used, and t-tests were applied. Categorical data were presented as frequency (n) and percentage (%), and the chi-square (χ^2^) test was used for comparison.

## RESULTS

### Transvaginal sonography and magnetic resonance imaging diagnostic results

Among 102 patients clinically suspected of early CSP, 80 were confirmed to have CSP by the reference standard (pathological or definitive clinical diagnosis). TVS correctly identified 67 of these 80 CSP cases, yielding a sensitivity of 83.75%, whereas MRI detected 75 out of 80 cases, corresponding to a sensitivity of 93.75%. MRI also achieved a higher overall diagnostic accuracy, correctly classifying 94 out of 102 patients (92.16%) compared to 82 out of 102 (80.39%) with TVS. The differences in sensitivity and accuracy between MRI and TVS were statistically significant (χ^2^=4.006, p=0.045 for sensitivity; χ^2^=5.961, p=0.015 for accuracy). In contrast, there was no significant difference between MRI and TVS in specificity (86.36 vs. 68.18%, χ^2^=2.071, p=0.150). Similarly, the positive predictive value (PPV) of MRI (96.15 vs. 90.54% for TVS) and the negative predictive value (NPV) (79.17 vs. 53.57%) did not differ significantly between the two modalities (PPV: χ^2^=1.141, p=0.286; NPV: χ^2^=0.438, p=0.508).

### Comparison of the coincidence rate of transvaginal sonography, magnetic resonance imaging, and pathological results

Using surgical pathology findings as the gold standard, MRI demonstrated higher detection rates than TVS for several key pathological features of CSP. MRI was significantly more effective in detecting intrauterine hematomas, identifying this finding in 41 out of 45 cases (91.11%) compared to 29 out of 45 cases (64.44%) with TVS (χ^2^=9.257, p=0.002). MRI also outperformed TVS in detecting invasion of the myometrium by trophoblastic villi (90.91 vs. 66.67%; χ^2^=5.802, p=0.033). Furthermore, MRI identified all instances of villi invading the bladder and/or cervix (five out of five cases, 100%), whereas TVS detected none of these cases (0/5, 0%), a difference that was statistically significant (p=0.008 by Fisher's exact test). By contrast, both MRI and TVS showed similarly high detection rates for the presence of a gestational sac (95.77 vs. 90.14%, respectively), and this difference was not statistically significant (χ^2^=1.721, p=0.190).

### Transvaginal sonography and magnetic resonance imaging clinical classification detection results

Postoperative pathology results showed that among 80 CSP patients, 32 cases were type I, 34 cases were type II, and 14 cases were type III. TVS detected the subtypes with an accuracy of 71.25% (57/80), while MRI detected the subtypes with an accuracy of 91.25% (73/80). The difference in detection accuracy between TVS and MRI was statistically significant (χ^2^=10.503, p<0.05) (Table 1).

**Table 1:** Detection results of clinical classification by transvaginal sonography and magnetic resonance imaging.

Group		Clinical gold standard classification				Total (n=102)
		Type I (n=32)	Type II (n=34)	Type III (n=14)	Negative (n=22)	
TVS	Type I	17	3	0	7	27
	Type II	2	30	4	0	36
	Type III	0	1	10	0	11
	Negative	13	0	0	15	28
MRI	Type I	26	0	0	3	29
	Type II	1	34	1	0	36
	Type III	0	0	13	0	13
	Negative	5	0	0	19	24

TVS: transvaginal sonography; MRI: magnetic resonance imaging.

### Imaging findings

**Figure 1:**
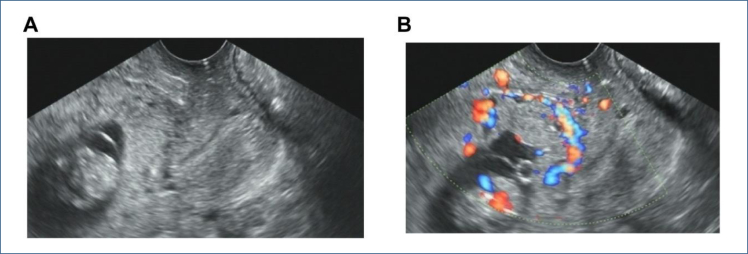
Transvaginal ultrasound image of a patient at 10 weeks and 3 days gestation with type III cesarean scar pregnancy. The ultrasound reveals that the placenta entirely overlies the internal cervical os, with the posterior edge of the placenta being entirely overlies the internal cervical os, with the posterior edge of the placenta being indistinguishable from the lower segment of the anterior uterine wall myometrium. There appears to be a discontinuity in the serosal layer (A). Color Doppler flow imaging demonstrates abundant blood flow signals localized to the scar site (B).

TVS: No gestational sac was identified within the uterine cavity or the cervical canal of the uterus. The gestational sac was implanted at the site of the prior cesarean section scar, with fetal bud or cardiac activity visible in part of the gestational sac. The myometrial layer between the gestational sac and the bladder was thinned or even absent. CDFI revealed high-velocity, low-resistance blood flow signals surrounding the gestational sac (Figures 1A, B).

**Figure 2:**
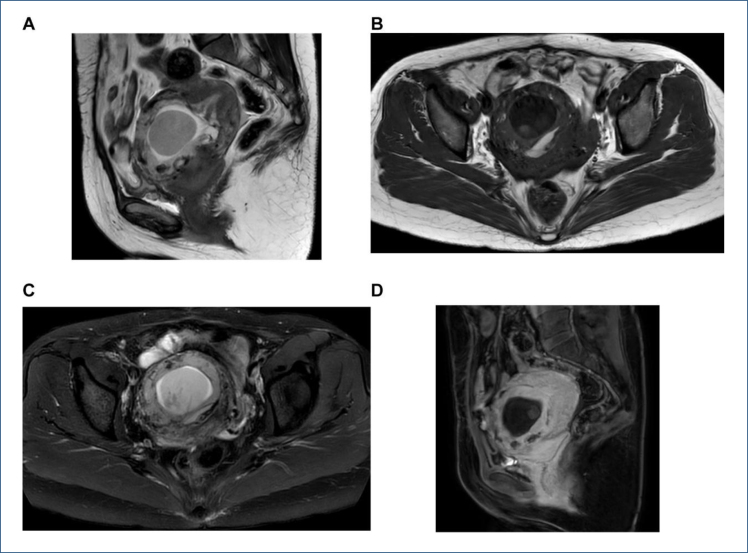
The aforementioned patient T2-weighted image (A), T2-weighted image-SPAIR (B), T1-weighted image (C), and Enhancement sequence (D). Magnetic resonance imaging shows a gestational sac within the uterine cavity, with placental implantation at the site of the previous cesarean section incision (as indicated by the red arrow). The placenta invades the myometrium reaching the serosal layer, with evidence of discontinuity. The boundary between the posterior wall of the bladder and the anterior uterine wall is indistinct.

MRI: The cesarean section scar in the lower anterior wall of uterus showed equal or low signal on T1WI and low signal on T2WI, and slight enhancement or no enhancement after enhancement. The scar area of the lower anterior wall of the uterus can be seen like a round cystic or cystic-solid gestational sac shadow. A typical gestational sac presents as a trilaminar structure. The innermost layer is the germ or fetal structure, T1WI is equal or slightly lower signal, and T2WI is slightly lower signal. The middle layer is the extraembryonic body cavity, the T1WI is mostly low signal, the complicated hemorrhage is high signal, and the T2WI is mostly high signal. The outermost layer is the villous structure, T1WI shows equal signal, and T2WI shows slightly higher signal. DWI showed limited diffusion and showed obvious or mild enhancement after enhancement. The myometrium at the scar site is thinned, partially interrupted, or partially absent (Figures 2 A–D).

## DISCUSSION

Cesarean scar pregnancy is a rare form of ectopic pregnancy characterized by implantation of the gestational sac at the site of a previous cesarean section scar. Although its pathogenesis remains unclear, poor healing of the uterine incision is widely considered a contributing factor^7^. The incidence of CSP is increasing. If not diagnosed and managed in a timely manner, it can lead to serious complications such as uterine rupture and massive hemorrhage, with patients even facing the risk of hysterectomy or death. The CSP classification is based on the depth and extent of implantation and its relationship with the uterine cavity^8^. Accurate classification is essential for individualized treatment planning and risk reduction. For instance, curettage may be appropriate for type I CSP under 8 weeks gestation, whereas type II or III CSP typically requires evacuation and scar repair. High-risk patients may benefit from preoperative methotrexate or uterine artery embolization. Therefore, early and accurate diagnosis and classification of CSP are crucial for formulating personalized treatment plans and improving patient outcomes.

Ultrasound, particularly TVS, is the first-line imaging modality for CSP diagnosis due to its ability to visualize the uterovesical space, assess myometrial thickness, and detect vascular signals around the gestational sac^9^. Compared to transabdominal ultrasound, TVS offers higher resolution and clearer anatomical delineation. However, TVS has limitations, especially in atypical cases. In patients with heavy bleeding or suspected miscarriage, chaotic intrauterine echoes may obscure the scar relationship, increasing the risk of misdiagnosis. In mass-type CSP, variable vascularity and irregular sonographic morphology can mimic conditions such as incomplete miscarriage or trophoblastic disease. Diagnostic accuracy is further influenced by the sonographer's experience and familiarity with CSP features.

Magnetic resonance imaging provides excellent soft tissue contrast and multiplanar imaging, offering distinct advantages in complex or equivocal cases. Our study demonstrated that MRI not only achieved higher sensitivity and accuracy than TVS in detecting CSP but also outperformed TVS in identifying intrauterine hemorrhage, myometrial invasion by villi, and invasion of adjacent structures such as the bladder and cervix. In our study, MRI demonstrated a significantly higher classification accuracy (91.25%) than TVS (71.25%) (p<0.05), confirming its utility in surgical planning. MRI's superior performance can be attributed to: (1) its ability to reconstruct images in multiple planes, offering clear visualization of the relationship between the gestational sac and the scar site; (2) its independence from patient body habitus or scar heterogeneity, providing detailed insights into the myometrium and surrounding anatomy^10^; and (3) its use of contrast-enhanced sequences when necessary, which improves visualization of tissue planes, scar defects, and invasion depth^11^. Despite its advantages, MRI is not infallible. In cases of incomplete miscarriage, residual tissue and clots may complicate interpretation. Without contrast enhancement, it may also be challenging to differentiate gestational tissues from other uterine lesions^12^. Moreover, diagnostic performance depends on the experience and expertise of the interpreting radiologist.

In conclusion, while TVS remains the first-line modality for CSP diagnosis, MRI serves as a valuable adjunct, especially in complex cases. Their combined application enhances diagnostic accuracy, informs treatment planning, and reduces the risk of adverse outcomes in women of reproductive age.

## CONCLUSION

MRI has higher sensitivity, accuracy, and diagnostic accuracy for different CSP classifications compared to TVS. It serves as an important supplementary tool to ultrasound, providing better guidance for clinicians to adopt individualized treatment for CSP patients, improving clinical treatment success rates, and reducing the risks of massive bleeding, hysterectomy, and even death in women of reproductive age.

## Data Availability

The datasets generated and/or analyzed during the current study are available from the corresponding author upon reasonable request.
